# Natural language processing data services for healthcare providers

**DOI:** 10.1186/s12911-024-02713-x

**Published:** 2024-11-26

**Authors:** Joshua Au Yeung, Anthony Shek, Thomas Searle, Zeljko Kraljevic, Vlad Dinu, Mart Ratas, Mohammad Al-Agil, Aleksandra Foy, Barbara Rafferty, Vitaliy Oliynyk, James T. Teo

**Affiliations:** 1 CogStack, Guys and St Thomas NHS Trust, London, UK; 2 Department of Biostatistics and Health Informatics, Institute of Psychiatry, Psychology and Neuroscience, King’s College London, London, UK; 3 Department of Clinical Neuroscience, Institute of Psychiatry, Psychology and Neuroscience, King’s College London, London, UK

**Keywords:** Natural language processing, Large language models, Bioinformatics, Machine learning, Electronic health records

## Abstract

**Purpose of Review:**

Embedding machine learning workflows into real-world hospital environments is essential to ensure model alignment with clinical workflows and real-world data. Many non-healthcare industries undergoing digital transformation have already developed data labelling and data quality management services as a vertically integrated business process.

**Recent Findings:**

In this paper, we describe our experiences developing and implementing a first-of-its-kind clinical NLP (natural language processing) service in the National Health Service, United Kingdom using parallel harmonised platforms. We report on our work developing clinical NLP resources and implementation framework to distil expert clinical knowledge into our NLP models. To date, we have amassed over 26,086 annotations spanning 556 SNOMED CT concepts working with secondary care specialties.

**Summary:**

Our integrated language modelling service has delivered numerous clinical and operational use-cases using named entity recognition (NER). Such services improve efficiency of healthcare delivery and drive downstream data-driven technologies. We believe it will only be a matter of time before NLP services become an integral part of healthcare providers.

**Supplementary Information:**

The online version contains supplementary material available at 10.1186/s12911-024-02713-x.

## Introduction

### Using natural language processing to “unlock” free-text information

With the adoption of electronic health records over the past decades, every patient encounter, investigation, diagnosis, and discussion are being recorded and stored. It is estimated that 80% of EHR(electronic health record) data exists in an unstructured format [1], this data consists of free-text documents filled with medical jargon, short-hand and abbreviations. To draw valuable insights and trends from clinical text data, it needs to be structured in a format digestible for computational models, only then can we deliver meaningful clinical impact.

Natural language processing (NLP) is a sub-group of artificial intelligence focused on the processing and analysis of text. To understand the complexity of medical language, most modern clinical NLP models undergo unsupervised training on large amounts of text data, subsequent fine-tuning and validation requires human labelled or “annotated” clinical text data. Annotating text data can be a time-consuming process and often very costly. Industries have used data-labelling services (like Amazon Mechanical Turk, Appen, Scale AI and Upwork etc.) where labelling and abstraction activity is outsourced to an external entity. Whilst outsourcing annotations can be pragmatic, it is unsuitable for clinical text. Firstly, clinical text contains protected health information and identifiable data, using third-party labelling services could risk significant data breach. Secondly, outsourcing annotators fail to leverage on the main benefits of labelling near the source - clinicians know the local context and jargon of what they are labelling, arguably they are also the best informed to understand and label clinical language that is used in medical documents. By leveraging domain experts to annotate clinical free-text at the source, we are able to curate a gold standard annotated text dataset which can be used to build, fine-tune or validate a healthcare-domain specific NLP model.

**Fig. 1:**
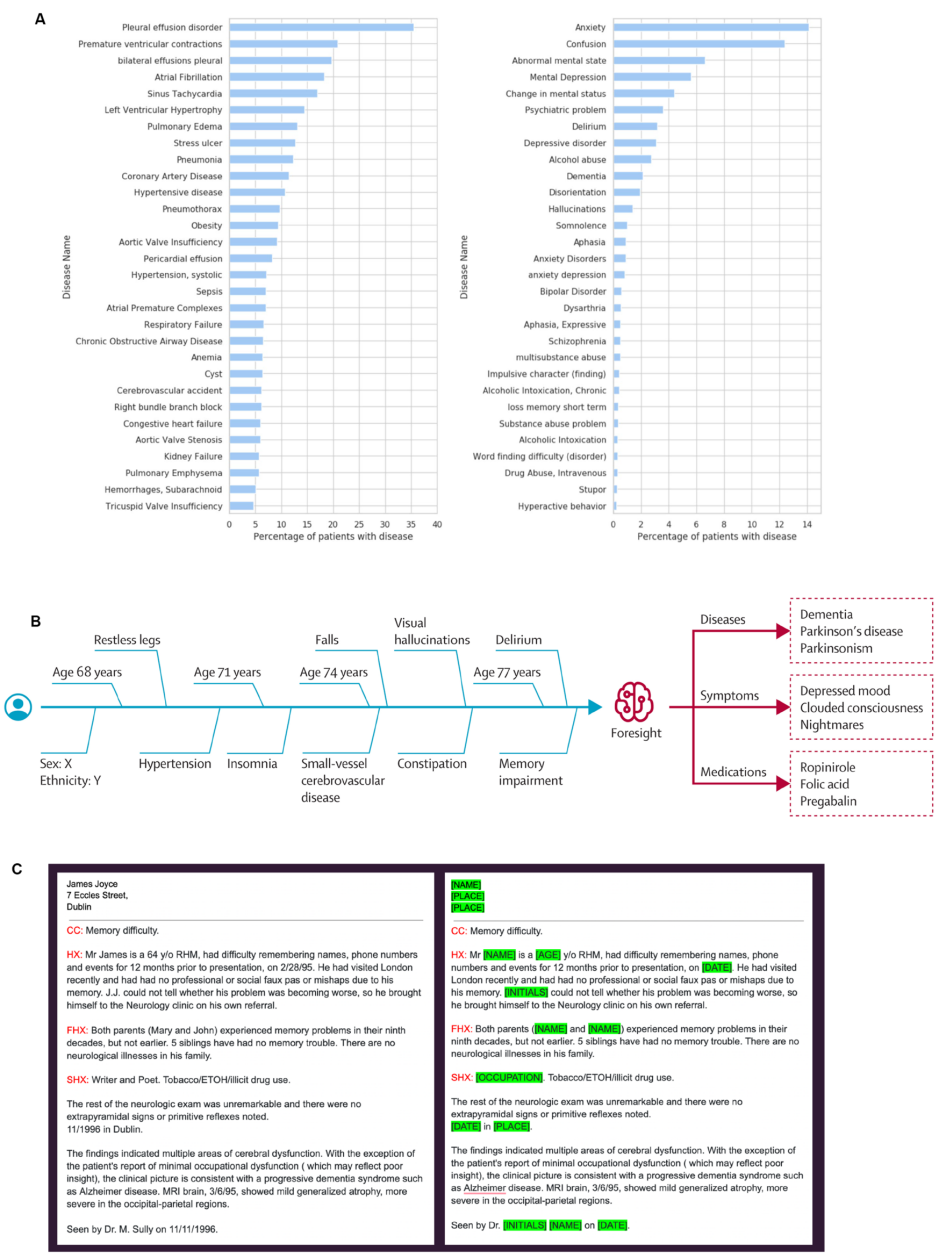
Natural language processing (NLP) case examples using MedCAT (medical concept annotation tool). (**a**) Surfacing vital clinical patient information to enable research and drive clinical insights (**b**) Visualising and forecasting patient timelines (**c**) personal health information redaction of electronic health records. (**a**) Using MedCAT to surface distribution of physical (Left) and mental disorders (Right) from MIMIC-III (an intensive care unit dataset) [8]. This approach was also used during the COVID-19 pandemic to elucidate risk factors and relationships with medications (ACE inhibitors) to help address international research questions [2]. (**b**) Visualising patient timelines using MedCAT enables us to better understand disease trajectories as well as public health planning. Patient timeline data can then be used to train an AI model (generative pretrained transformer) to predict the next probable clinical event. (Image from publication Zeljko et al.) [6] Creative Commons Attribution (CC BY 4.0). (**c**) Using MedCAT with clinician fine-tuning, we are able to redact personal health information to preserve patient privacy. We have de-identified and stored over 2 million free-text documents in King’s College Hospital using this approach, this enables safer data-sharing approaches for future research and operational projects [5]

**Fig. 2:**
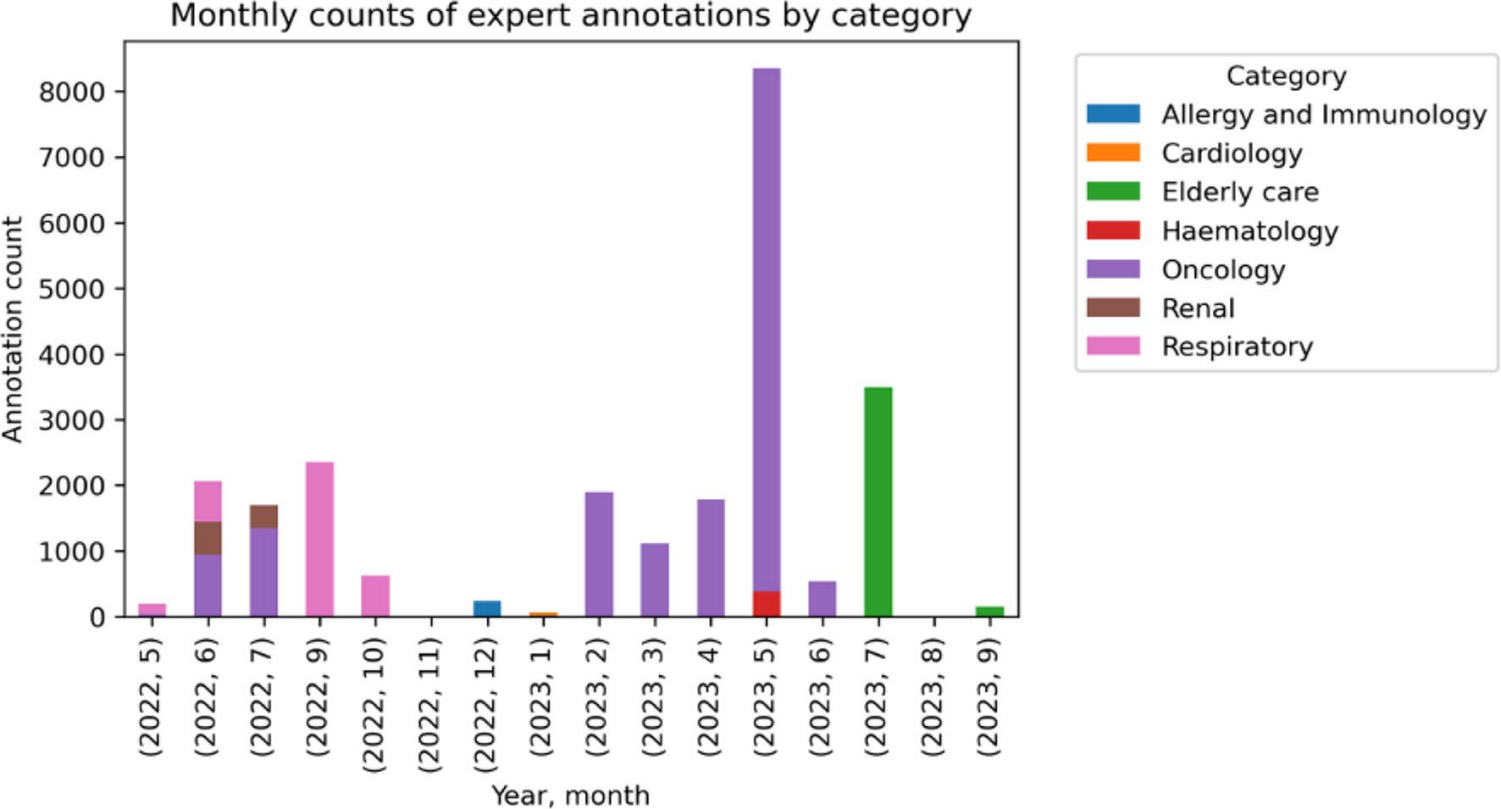
Graph showing monthly expert annotation counts over time (total 26086 annotations, spanning 556 SNOMED-CT concepts), coloured by secondary care department

We describe the experiences of embedding the first natural language processing service in the UK national health service (NHS) and instilling clinical knowledge from clinician annotators. We share our published work of how an NLP service can support projects in healthcare, our learnings, resources and experience using our NLP software MedCAT (Medical concept annotation tool) to uncover valuable insights [2], reveal clinical trends, automating time-consuming administrative tasks, clinical coding [3], monitor disease trends [4], data anonymisation [5], forecast patient trajectories [6], summarising hospital-wide health data [7] and more (Fig. 1). We share our clinical text annotation best-practice framework (supplementary A) to map clinical text to clinical classification systems, also known as “named entity recognition + linking” (NER + L), like ICD-10 or SNOMED-CT. To date, we have amassed over 26,086 annotations spanning 556 SNOMED CT concepts working with secondary care specialties (Fig. 2).

### The technology

**Fig. 3:**
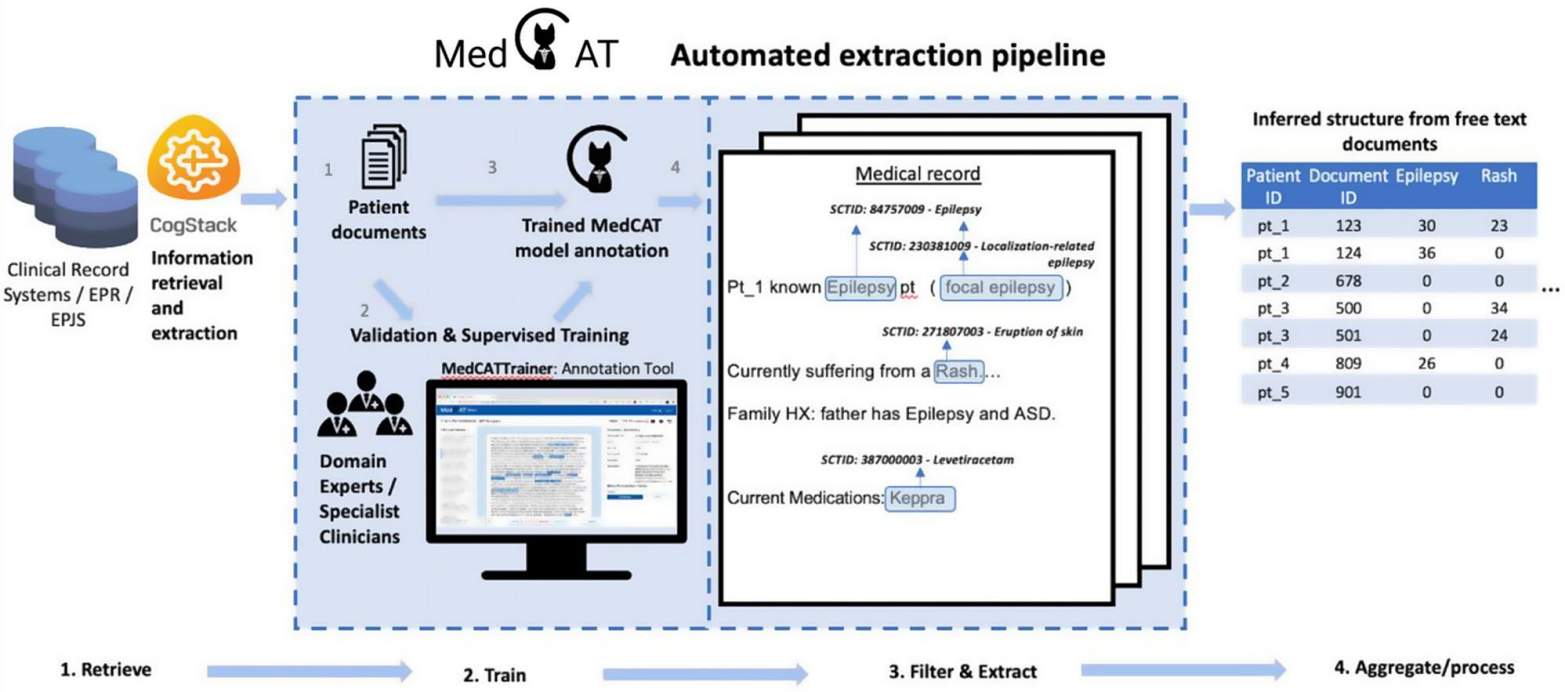
The NLP infrastructure - free text entries within multiple clinical record systems / electronic patient records are ingested and indexed into CogStack. Ingested documents can then be structured using MedCAT (medical concept annotation tool), an AI/NLP automated extraction pipeline for named entity recognition + linking. The code together with tutorials are available on GitHub (https://github.com/CogStack/MedCAT)

The CogStack platform is deployed across King’s College Hospital NHS trust, Guys and St’ Thomas’ NHS trust, and South London and Maudsley NHS Trust. The CogStack platform functions as a data lake that draws data (including free-text) from multiple electronic health records and other data sources [9]. Documents retrieved through CogStack are subsequently processed using the MedCAT (medical concept annotation toolkit), an NLP toolkit for named entity recognition + linking (NER + L) (Fig. 3) [10]. 

## Key learnings from implementation and delivery of a natural language processing service in the NHS

### Distilling and encoding domain-expert knowledge

Expert clinical knowledge amongst clinical experts combines knowledge about diseases, diagnostics, clinical decision-making, together with the implicit working knowledge of clinical workflows, styles of clinical practice and clinical intent. Distilling these forms of expert knowledge into a model that allows scalable application of this knowledge to large volumes of healthcare data. For NLP, this task is labelling and annotating text using the expert’s linguistic experience and contextual knowledge of clinical workflows and intent.

### Aligning team incentives to drive success

Successful projects require clinician engagement and time, in particular clinicians have to be actively committed to the task of annotating and the iterative process of correcting annotations, this can often be a repetitive and tedious task. Therefore, it is important to set clear objectives and milestones before the project is commenced. During the process, the NLP service should give annotators support and guidance in the annotation process to ensure annotation alignment between annotators. The most successful projects are ones where clinicians have identified a clear clinical or research problem that they wish to solve.

### Capturing annotations accurately and concisely

**Fig. 4:**
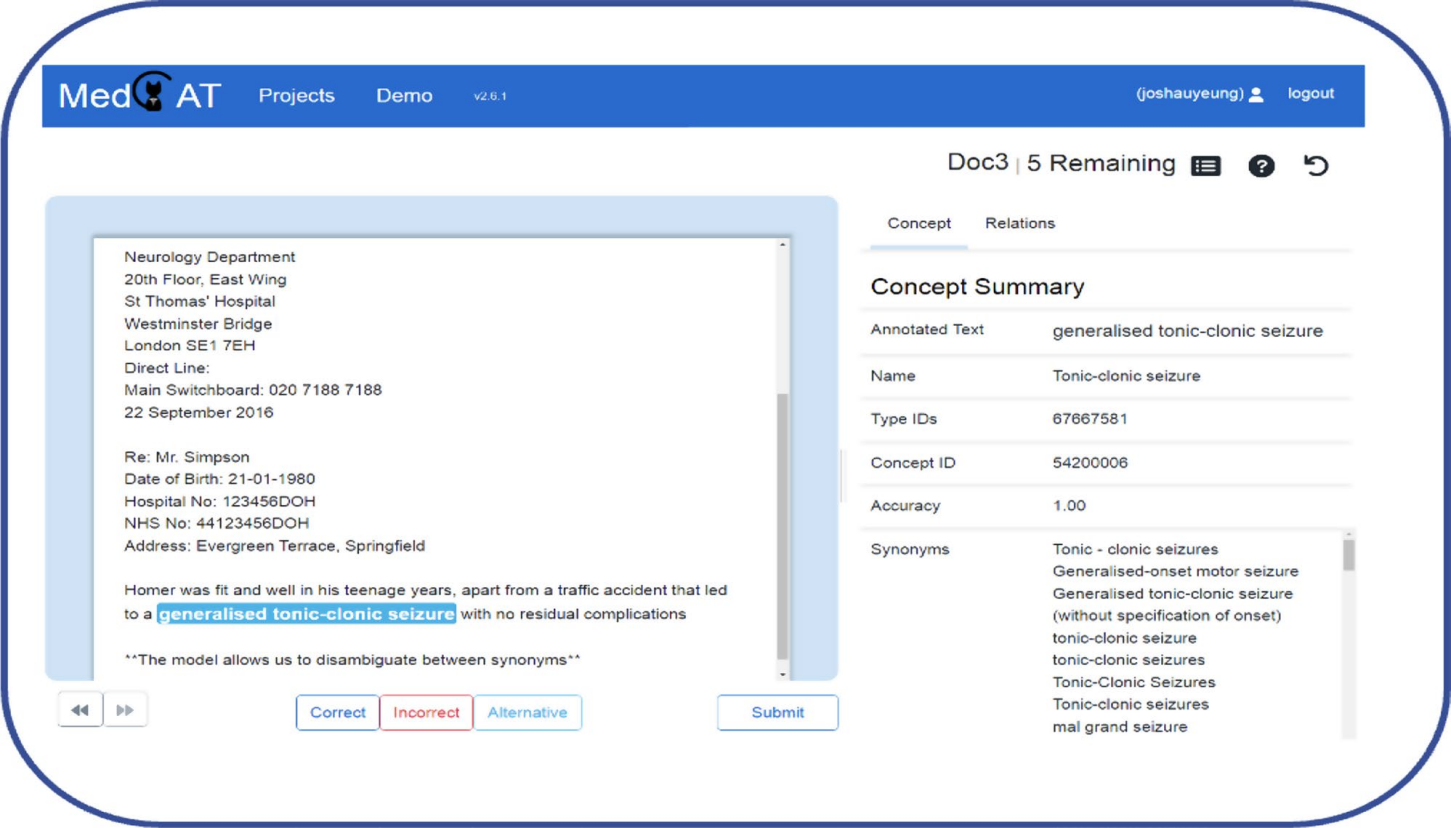
User interface of MedCAT trainer. Clinicians mark concepts or clinical codes detected by the model as correct, incorrect or offer an alternative annotation or manual annotation [11]

The MedCAT interface was designed by developers and researchers in conjunction with clinicians to make annotating intuitive [10]. The MedCAT trainer model uses an online learning process that recognises concepts being annotated and automatically flags up similar concepts in subsequent documents (Fig. 4) [11]. This is useful to reduce annotator fatigue, as well as reduce the incidence of a term being missed in a long span of text.

## A framework for implementing natural language processing as a service

**Fig. 5:**
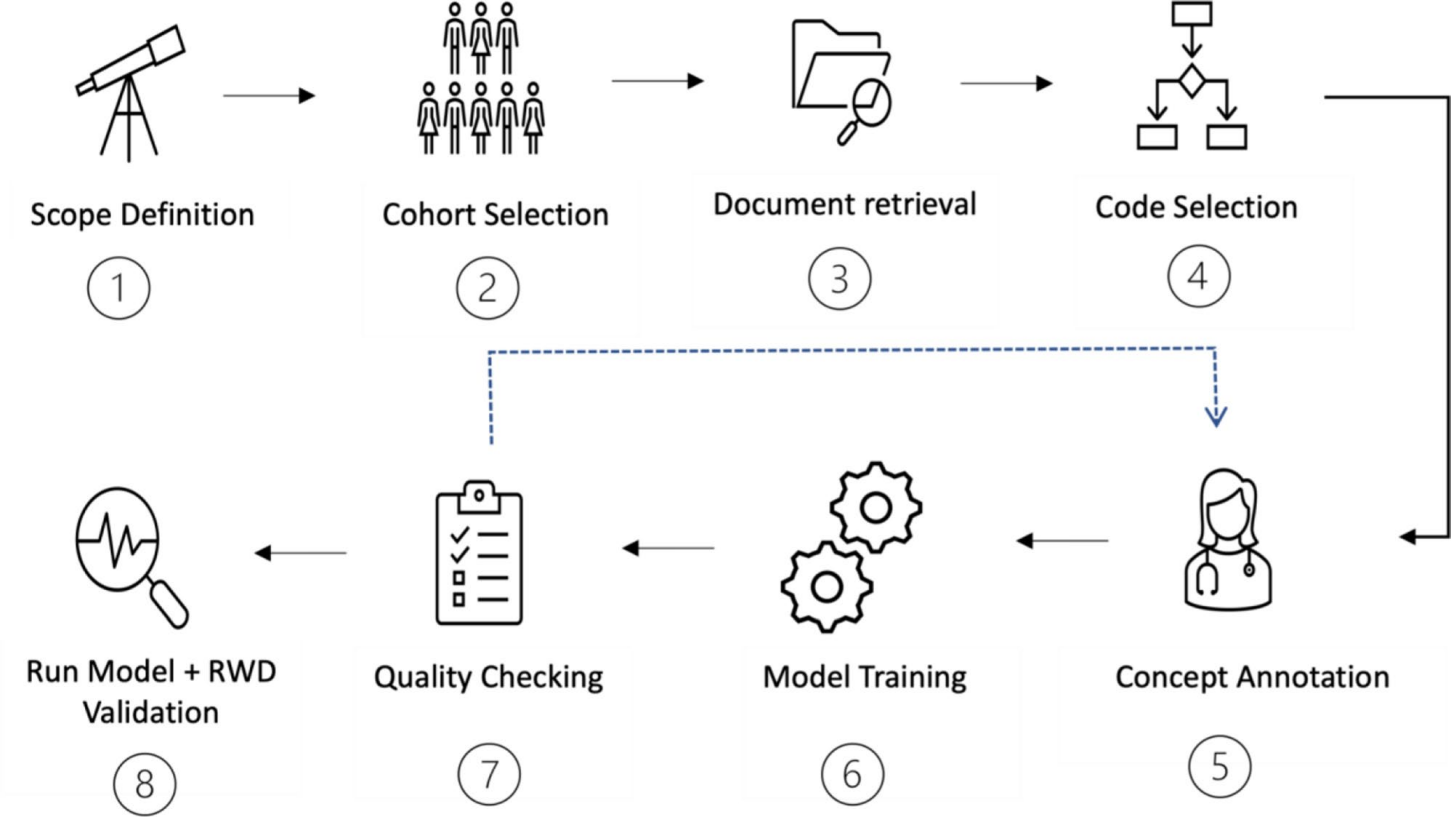
A framework for implementing natural language processing to generate real world data (RWD). In steps 5–7 we use a training-correction cycle to optimise the quality of annotations and model training

Below we highlight a NLP implementation framework from scope definition, annotation guidelines, to model deployment and real-world validation. The annotation framework establish an annotation standard that can be used to obtain consistent, high-quality medical annotations that is then used for model building, fine-tuning and validation (Fig. 5).

### 1. Scope definition

The first step is identifying the scope and nature of the project- is it a clinical audit or service evaluation, a research project or an operational evaluation? Appropriate approvals should be obtained prior to commencement of the project.

### 2. Cohort selection

In this step, we select the **patient** or **population** group of interest, and optionally the **comparison** or **control** group. Clinical departments or research teams may opt to supply an existing curated patient list that has been collected manually or exist in a database. Alternatively, the entirety of the patient text records can be coded using an NLP-model approach discussed below [7], the cohort can then be filtered based on clinical codes of interest.

### 3. Document retrieval

The next step is to retrieve relevant documents and meta-data from the patient cohort that helps address the project scope/ aim. Important considerations include:

- Document selection – Which documents does the data sit within the EHR?
- Meta-data selection - depending on what is captured in EHRs, we may wish to retrieve specific structured meta-data to help address our scope e.g. medications, department numbers, allergies, investigation orders and results.
- Timeframe- What timeframe of documents do we wish to extract (all historical patient documents, or focus on focus on a defined time period)?
- Pre-processing and data privacy [12] - redaction/ anonymisation of sensitive patient data, pre-processing free text to reduce noise.

### 4. Code selection / creation

This step involves identifying the information, or **“named entities”**, that we wish to extract from the clinical free text. The goal is to take free-text and structure it into a machine-readable format. We are able to structure free-text according to the selected concepts- usually clinical codes are used (such as SNOMED-CT, ICD-10, UMLS etc.). We can map synonymous mentions of “atrial fibrillation”, “afib” or “AF” to the SNOMED CT code − *49,436,004*,* Atrial fibrillation (disorder).*

It is particularly important to decide on the **depth** of the selected codes. For example, if you are interested in diabetes mellitus as a concept- Are you mapping all mentions of diabetes mellitus as a general disease? Do you wish to annotate detailed classification codes to distinguish between type 1 and type 2 diabetes, or complications e.g. diabetic retinopathy, diabetic nephropathy.

### 5. Concept annotation

This step is often the most important and labour intensive, annotating involves working with expert clinicians to label clinical free text to create a ground truth dataset. The goal of annotation is to consistently label concepts within free text to classification codes, this creates a dataset that the NLP model can be trained on.

For example in the phrase: “This patient has a bronchial adenocarcinoma affecting the left lower lobe. The cancer was recently diagnosed in Oct 2023”. In this scenario, if one labelled “cancer” as the concept < lung cancer>, the annotation would be project specific while if one labelled “cancer” as the concept < malignancy>, the annotation would be generalisable.

Consistency between annotators is essential for model learning, when there is more than one annotator, it is important to evaluate inter-annotator agreement; all annotators are given an overlapping set of documents to annotate, and the degree of agreement between annotations are calculated. Additional contextual concept information can be added as meta-annotations (supplementary A).

### 6. and 7. Model training and quality checking

The next step is to train and optimise the performance of the NLP model, and perform subsequent quality checking [12]. We use an iterative training-correction cycle to validate that extracted annotations are correct and optimised [12]. The process is as follows:

I. We split the clinician documents annotations into a train and test set (classically at 80:20 split). We then train an NLP model using our training set.
II. We run the NLP model over the clinician annotated documents and compare model outputs to clinician annotated outputs.
III. Find all false positive (FP) and false negative (FN) examples between NLP model and clinician annotators.
IV. Manually review each FP and FN, if the FP/FN is a mistake or omission from the annotator, we can amend the annotations and return to step (I). If there are no correctable FP/FNs, we continue to step (V).
V. Once all FP/FN are corrected, the final iteration of our trained NLP model is our optimised model. The performance of this model can now be assessed against our test set and NER + L performance calculated.

### 8. Run model + Real world-validation

The fine-tuned NLP model is then ran over the entire corpus of documents of interest to produce an aggregated tally of mentions of each medical concept within each document, which we refer to as “MedCAT mentions”. We can aggregate all relevant MedCAT mentions for each patient overtime. In a similar process to a clinician reading through and interpreting a patient’s previous history, we need to determine a threshold of MedCAT mentions where we can infer the true presence of a concept with a degree of certainty. For example, a single mention of the concept < hypertension > in an individual’s entire document history may be noise rather than signal- they may have suffered from a one-off hypertensive episode driven by anxiety or white-coat hypertension, rather than chronic hypertension.

**Fig. 6:**
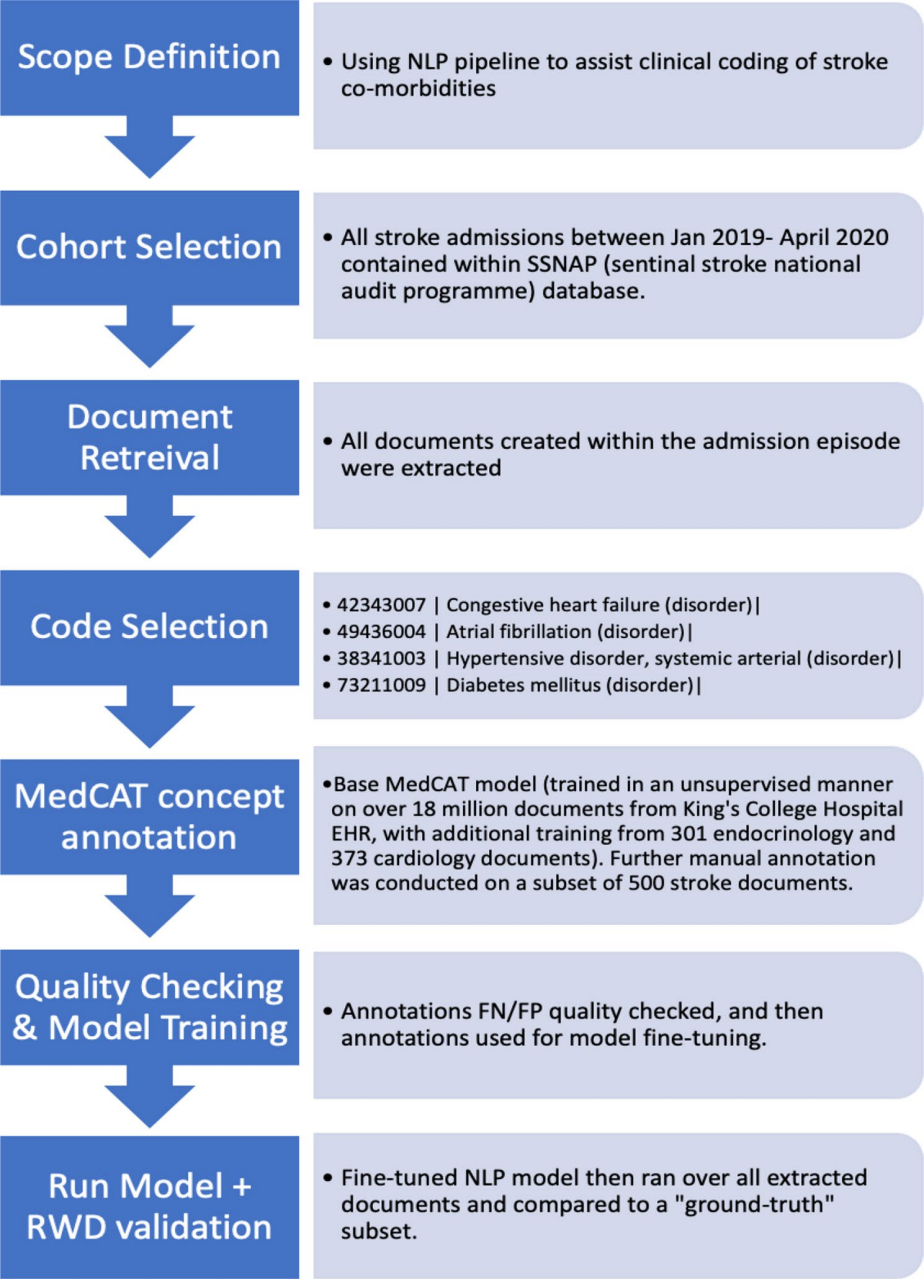
Example of using the MedCAT NLP pipeline for AI-assisted clinical coding for audit purposes that has been published. Clinical coding is the task of transforming medical information in a patient’s health records into structured codes for downstream analysis and organisational planning, an NHS coding department of around 25–30 coders usually codes over 20,000 cases per month [13]. Our approach could save clinical coders significant manual labour and increase performance and depth of coding [3]

Once a threshold has been set, each concept or disease in a patient then needs to be validated to a “ground truth” dataset in the real world. The ground-truth dataset is created by clinicians manually reading through patients’ documents and documenting the presence or absence of the concepts of interest. This final process allows confirmation and validation of the medCAT pipeline for concept or disease inference. An example is shown in Fig. 6 of applying this framework to a clinical audit.

## Future directions - building a large language model for the UK national health service (NHS)

We have demonstrated how to leverage annotators at the source to distil expert clinical knowledge into our NLP models. With the recent popularisation of large language models (LLM), there has been significant excitement on the potential of LLMs in healthcare [14, 15]. Here we describe future directions of distilling expert clinical knowledge to fine-tune a foundational large language model grounded in healthcare.

**Fig. 7:**
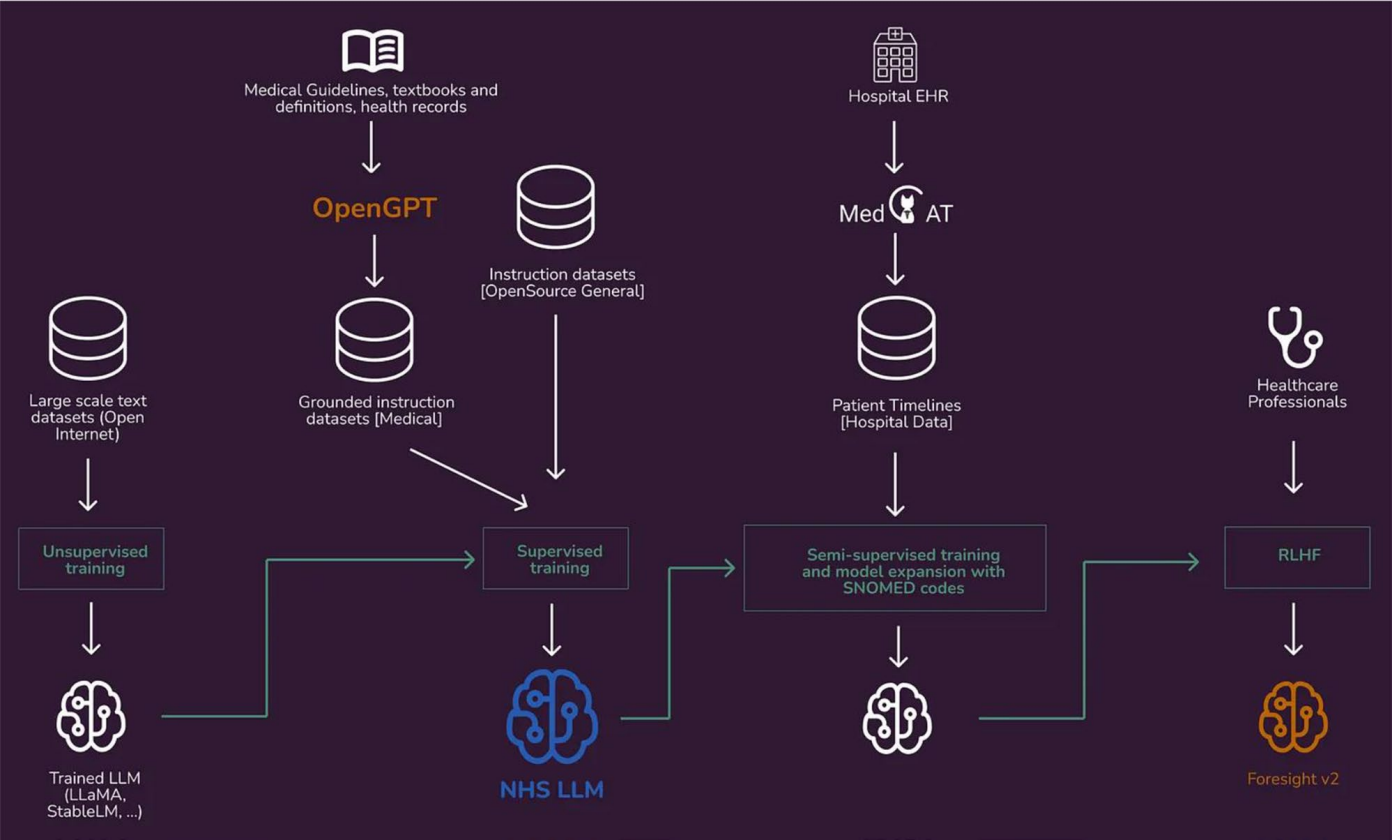
A diagram demonstrating an overview to fine-tuning large language models (LLMs) by leveraging clinician annotators and grounding in medical datasets

One key step towards a grounded foundational model is to go beyond published clinical knowledge and instil real-world healthcare knowledge. We demonstrate a novel approach recently published from Zeljko et al. on how we can leverage clinician-finetuned MedCAT to extract clinical annotations and construct patient timelines [6]. These timelines can be seen foundational data for foundational models that is distilled real-world clinical knowledge derived from electronic health records. The next step is to align the LLM with clinician intent and preference to overcome limitations such as hallucinations, inconsistent outputs and misalignment [16–18]. One approach to this is to train a reward model through reinforcement learning with human (clinician) feedback (RLHF) where clinician annotators can rank and re-write LLM outputs. Generating a reward model through clinician feedback is a relatively new field, and one that future works should explore.

As a proof of concept, we have built NHS-LLM based on the foundational model of LLaMA 13B(Meta) [19] that was further grounded through instruction tuning with datasets such as the NHS UK conditions website and NICE guidelines (Fig. 7). From a qualitative perspective, NHS-LLM produces more grounded outputs compared to proprietary models such as ChatGPT and GPT-4. We aim to build on this approach to create an LLM that can be used for clinical settings, one that is less prone to hallucinations and more grounded in facts [20] (Fig. 7). 

## Conclusion

**Fig. 8:**
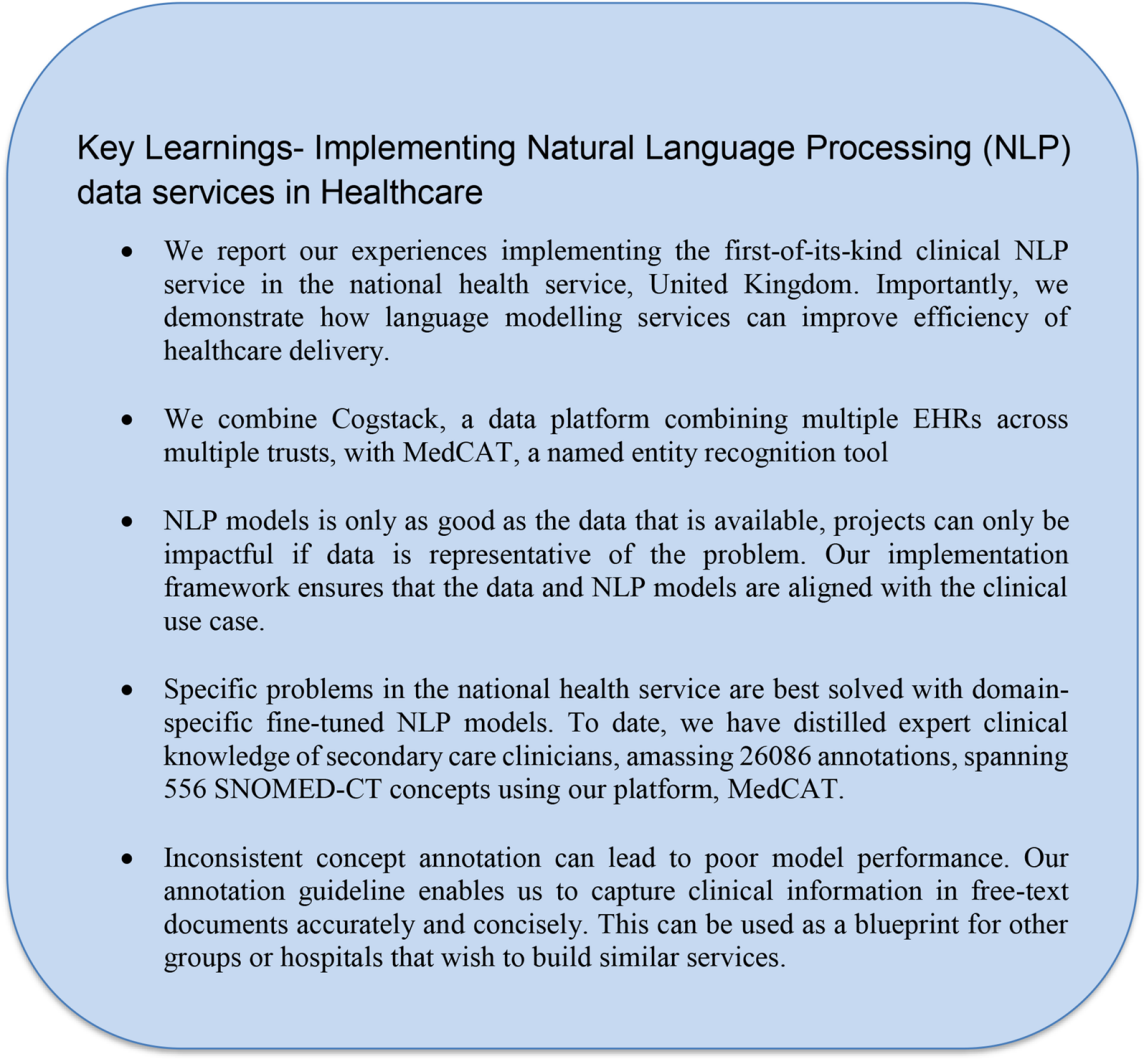
A table summarising key learnings from implementing the first-of-its-kind Natural Language Processing data service in the National health service (NHS). The learnings are combined perspectives from our multidisciplinary team including clinicians, developers, and researchers

We have described our experiences as the first embedded natural language processing service operating within the NHS, using approaches such as named entity recognition to support projects and “unlock” data in the electronic health records. As an internal healthcare service, we are able to navigate traditional barriers to AI in healthcare such as data access, privacy, access to clinician annotators at the source, in turn we are able to deliver service-changing impact. We have laid out the blueprint to organise and leverage clinician expertise to annotate and collaboratively instil their knowledge into NLP models Fig. 8. We believe it will only be a matter of time before NLP services become an integral part of healthcare providers.

## Electronic supplementary material

Below is the link to the electronic supplementary material.

Supplementary Material 1

## Data Availability

Project name: Medical concept annotation tool (MedCAT). Project home page: https://github.com/CogStack/MedCAT. Operating system(s): Windows, Linux, MacOS. Programming language: Python. Other requirements: Python 3.0 or higher. License: Elastic 2.0. Any restrictions to use by non-academics: The licens or grants you a non-exclusive, royalty-free, worldwide, non-sublicensable, non-transferable license to use, copy, distribute, makeavailable, and prepare derivative works of the software. Details of each case subject to the limitations and conditions as specified in the license document.
