# Supplementary material for: Natural language processing data services for healthcare providers
Source: BMC Med Inform Decis Mak. 2024 Nov 26;24:356. doi: 10.1186/s12911-024-02713-x (PMC11590340; doi:10.1186/s12911-024-02713-x)
Supplement: Supplementary file 1 — Supplementary Material 1 [file 12911_2024_2713_MOESM1_ESM.docx]

### Supplementary A - Annotation Guidelines

**Annotation Tool**

The tool being used for this annotation task is [MedCAT](https://github.com/CogStack/MedCAT/tree/master/tutorial)

Available - <https://github.com/CogStack/MedCAT>

### **Annotation Steps**

The annotation process is as follows (please do not skip documents, annotate one by one without jumping around). As can be seen from the flow-chart if something does not pass a step (e.g. Diagnosis is NO) there is no need to do any other meta-annotations (Status, Time), you can ignore them and **immediately go to the next annotation**:


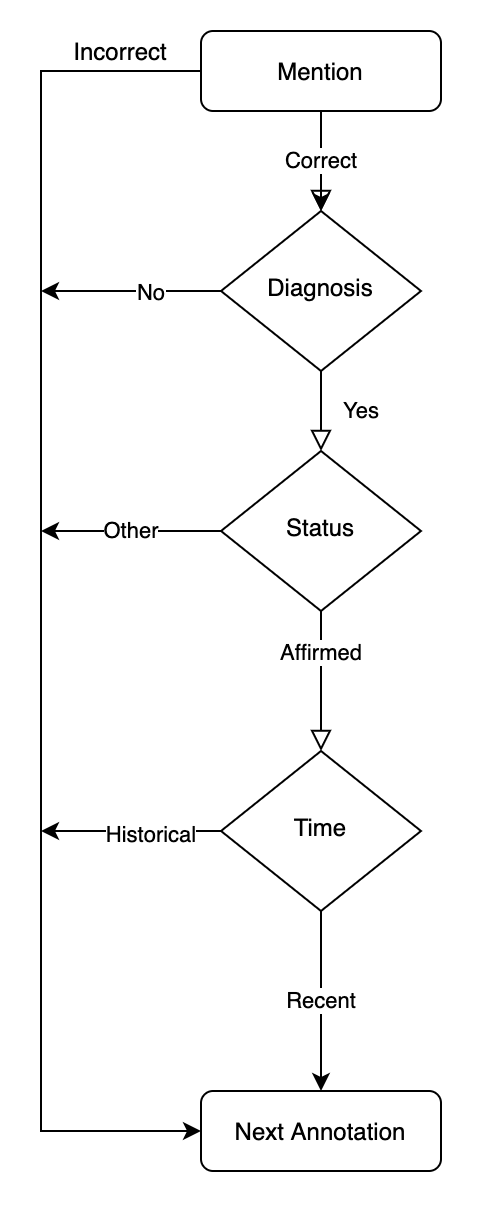


**The 4 steps are:**

1. Mention level correctness (Correct/Incorrect): Is the detected text (in blue) related to the Concept to which it refers (top right in Figure 1). If this is correct, go to the next step in the flow chart, if it is not correct - go immediately to the next annotation in that document (automatic), or submit this document if there is nothing else to check inside of it.

| Text | Detected concept | Mention Level Correctness |
| --- | --- | --- |
| The epilepsy nurse was... | Epilepsy | Correct |
| The patient had a fit... | Seizure | Correct |
| The pants did not fit... | Seizure | Incorrect |
| The diabetic clinic was... | Diabetes | Correct |
| Her mother is a diabetic | Diabetes | Correct |

1. Diagnosis (Yes/No): Is this comorbidity part of a **patient’s** diagnosis even if self-reported or positive/negative/hypothetical (i.e. does the annotated text refer to a diagnosis regardless of whether the patient has it or not). If it refers to a family member, it is not a diagnosis for the patient (annotate as No). We are ignoring drugs as proxies (even if the drug contains the name of the comorbidity), same for tests - even if the test is positive, we are not using it - it is not a diagnosis. There is an exception to the test rule - comment by Rob:
   1. “The blood results show <comorbidity>” - Hmm, it's quite borderline. The 'show' statement implies a diagnosis. It's one of those ones that's a little entity-dependent. For example if the diagnosis of interest was 'hyponatraemia' then that's something that only a blood test shows. Likewise 'the scan/biopsy shows cancer' is essentially a diagnostic statement. I would be inclined to include it because of the 'show' word (or equivalent).

| Text | Diagnosis |
| --- | --- |
| The epilepsy nurse was... | NO |
| The patient had a fit... | YES |
| The pants did not fit... | NA |
| The diabetic clinic was... | NO |
| The patient was taking diabetic medications... | YES |
| I have some pain in my legs, maybe it is diabetic neuropathy. | YES (status: Other) |
| I think I have cancer. | YES |
| The test for CoVid-19 was positive | NO |
| Tested for Salmonella, ... | NO |
| Blood results show <comorbidity> | YES (read the comment) |
| Insulin pen for diabetes.. | NO |
| Patient’s diabetes care... | YES |
| History of diabetes management is... | NO |
| Her mother is a diabetic | NO |
| Diagnosis of diabetes insipidus | NO (correct at mention level) |

1. Status (Affirmed/Other): Is the detected comorbidity Affirmed (patient has it) or Other (patient thinks he has something, patient does not have something …)

| Text | Status |
| --- | --- |
| The patient had a fit... | Affirmed |
| The diabetic clinic was... | NA |
| I have some pain in my legs, maybe it is diabetic neuropathy. | Other |
| I think I have cancer. | Other |

1. Time (Recent/Historical): Based on the context only, we are to say is this something that is recent or is it expressed as PMH (Past Medical History). Please try to select this as often as possible (it is very tempting to just ignore / leave in NA). In most cases a comorbidity will be recent, even if in the text it is not expressed in that way. We will only say historical if explicitly written in the sentence that it is historical (e.g. He had a strong a long time ago; PMH of ...)

| Text | Status |
| --- | --- |
| The patient had a fit today... | Current |
| PMH: DM2 | Historical |
| The patient had a fit two years ago... | Historical |
| The patient is diabetic (or has diabetes) | Current |
| Patient (diabetic) was... | Current |

Click on “Submit” when done with all the annotations in the document, to confirm your annotation for this document and move on to the next one.

Note: You won’t be able to “submit” until you address all the pre-annotated mentions.

**More examples:**

- We are not using drugs as proxies
- We accept self-reported diseases Status=Affirmed - unless the self-report is obviously wrong/delusional then Status=Other
- Test results: we are completely ignoring all test results and accepting something only if it is part of a diagnosis (here it means explicitly stated the patient has a comorbidity). An exception to this rule is the comment above by Rob:
  - “The blood results show <comorbidity>” - Hmm, it's quite borderline. The 'show' statement implies a diagnosis. It's one of those ones that's a little entity-dependent. For example if the diagnosis of interest was 'hyponatraemia' then that's something that only a blood test shows. Likewise 'the scan/biopsy shows cancer' is essentially a diagnostic statement. I would be inclined to include it because of the 'show' word (or equivalent).

| **Example** | **Concept** | **Diagnosis** | **Status** | **Time** |
| --- | --- | --- | --- | --- |
| Patient has epilepsy | epilepsy | YES | Affirmed | Current |
| Patient does not have epilepsy | epilepsy | YES | Other | NA |
| Patient says that he was told that this was polio | Polio | YES | Other | NA |
| He says he applied eczema cream twice daily | eczema | NO | NA | NA |
| He mentioned having chicken pox | Chicken pox | YES | Context-dependent | Context-dependent |
| He mentioned having smallpox | Smallpox | YES | Context-dependent | Context-dependent |
| Recent stool sample  Salmonella, shigella and campylobacter - not isolated | shigella | NO | NA | NA |
| Patient reports he has polio | polio | YES | Context-dependent | Context-dependent |
| Patient was seen by epilepsy nurse | epilepsy | NO | NA | NA |
| Patient could have epilepsy | epilepsy | YES | Other | NA |
| Patient thinks he has cancer | cancer | YES | Other | NA |
| Patient advised to receive vaccinations for hepatitis | hepatitis | NO | NA | NA |
| patient accepted an RCT for diphtheria | diphtheria | NO | NA | NA |
| “...patient has been diagnosed with diabetes mellitus…” | Correct | Yes | Affirmed | Unknown |
| “....no history of diabetes….” | Correct | Yes | Other | Unknown |
| “...his mother is on diabetes medications…” | Correct | No | NA | NA |
| “Diabetic Annual Review Letter..” | Correct | No | NA | NA |
| “..higher risk of developing diabetes..” | Correct | No | NA | NA |
| “..thank you for your ongoing monitor/management of his/her diabetes as per NICE guidelines for Schizophrenia" | Correct | Yes | Affirmed | Unknown |
| “..she is borderline diabetic” | Correct | NoYes | NA | NA |
| "He stopped taking medications for his diabetes" | Correct | Yes | Affirmed | NA |
| "Patient has little insight into his diabetes" | Correct | Yes | Affirmed | Current |
| “..need to avoid diabetes..” | Correct | No | NA | NA |
| Patient concerned about increased frequency and thirst and having diabetes | Correct | Yes | Other | NA |
| Prescribed Metformin 500mg tds for **diabetes** | Correct | Yes | Affirmed |  |
| ...review **diabetes** | Correct | Yes | Affirmed |  |
| 'mental state is improved, as is her diabetes', | Correct | Yes | Affirmed |  |
| saw X and discussed how to monitor his **diabetes** | Correct | Yes | Affirmed | Current |
| ..is on treatment for **diabetes** | Yes | Yes | Affirmed | Current |
| He preferred to be on oral **diabetic** tablets | Yes | No | NA | NA |
| GP for his review of **diabetes** due to raised BM | Yes | No | NA | NA |
| He was given advice on maintaining diabetic diet and healthy eating | Yes | No | NA | NA |
| CP2-**Diabetic** diet adhered to | Yes | No | NA | NA |
| ..he also agrees to see a **diabetic** nurse. | Yes | No | NA | NA |
| GP informed me that he is due for a diabetes review in January | Yes | No | NA | NA |
| His **diabetic** care plan | Yes | Yes | Affirmed |  |
| his routine **diabetic** review. Advised to attend physical health and **diabetic** reviews | Yes | Yes | Affirmed |  |
| his increase in **diabetic** medication Gliclazide | Yes |  |  |  |
| diabetic foot screenings and eye screenings | Yes | Yes | Affirmed |  |

**Glossary**

**Annotation**: A note by means of a comment added to a meaningful word from the sentence. For example, to annotate mentions of diseases with SNOMED Concept IDs within a sentence: “The patient has no family history of epilepsy” {Epilepsy, SCTID: S-84757009}. This denotes that the underlined word is epilepsy within the SNOMED-CT under SCTID: S- 84757009.

**Meta-annotation**: A meta-annotation is an annotation that can be applied to another pre- existing annotation. A meta-annotation is usually used to provide further information of the context of the pre-existing annotation. For example: “The patient has a negative family history of epilepsy” {Epilepsy, Experiencer: Family, Presence: Negative} This denotes that the annotation epilepsy is a negative mention and is mentioned in a context which refers to the Family.
